# Enhancing physical activity levels in 9–11-year-old children of varied genders: strategies leveraging parental exercise consciousness

**DOI:** 10.3389/fpsyg.2025.1407090

**Published:** 2025-02-06

**Authors:** Chao Song, Sha Ge

**Affiliations:** College of Sports Science, Tianjin Normal University, Tianjin, China

**Keywords:** physical activity, exercise consciousness, children, parental style, age, gender

## Abstract

**Objective:**

This study aimed to explore the predictive associations of parental exercise consciousness with the physical activity (PA) status and characteristics of 9–11-year-old children, in an attempt to identify effective strategies for addressing PA insufficiency in children from a parental standpoint.

**Methods:**

The PA rating scale-3 and the Chinese Civic Exercise Consciousness Questionnaire were used to measure primary school students’ PA and their parents’ exercise consciousness from 361 families in Tianjin, China. The dimensions of exercise consciousness were cognition and identity (CI), sentiment and intention (SI), attitude and willingness (AW), and rights and responsibilities (RR) dimensions. Descriptive statistics, ANOVA with post-hoc test analysis, correlation analysis, and hierarchical regression analysis were performed.

**Results:**

First, children’s PA increased with age, with boys significantly exceeding girls (*p* < 0.01). Significant gender differences were observed in both PA intensity and frequency (boys higher than girls, *p* < 0.01), although this was not consistent across age groups. Variations in PA duration and frequency showed significant age-related changes (*p* < 0.01), whereas intensity showed significant changes at older ages (*p* < 0.01). Second, we observed moderate to above-average level of parental exercise consciousness, and a significantly positive correlation between parental exercise consciousness and children’s PA levels (*α* = 0.601, *p* < 0.01); meanwhile, all four dimensions of parental exercise consciousness showed varying degrees of positive correlations with children’s PA intensity, duration, and frequency (*p* < 0.01). Third, stratified linear regression analysis showed the varied predictive associations of the different dimensions of parental exercise consciousness with children’s PA intensity, duration, and frequency. SI and AW significantly predictively associated with PA levels across different age groups and genders (*p* < 0.05).

**Conclusion:**

Age and gender showed crucial associations with children’s PA levels, resulting in distinct preferences and behaviors between boys and girls across various age groups. Parental exercise consciousness of exercise positively associated with the intensity, frequency, and overall volume of children’s PA. Enhancing parental consciousness of exercise-related emotions, intentions, attitudes, and willingness may effectively associate with enhancements in the intensity, duration, and frequency of their children’s PA, ultimately promoting greater engagement in physical exercise among the youth.

## 1 Introduction

An energetic and consistently evolving family environment that fosters sports activities is essential for the sustainable and healthy advancement of social health, with PA serving as a significant metric of health (Van Der Horst et al., 2007; Prüss-Ustün et al., 2017; Lee et al., 2018; Parker et al., 2019). Physical inactivity is recognized by the World Health Organization as the fourth highest risk factor for mortality worldwide. It is well documented that PA benefits body composition, cardiometabolic health, mental wellbeing, and physical fitness, and yet a sizable portion of adolescents lack engagement in related activities, representing a public health concern (Goran et al., 1999; Poitras et al., 2016; Biddle et al., 2019). Research indicates that PA engagement tends to diminish as individuals progress from adolescence to adulthood (Nelson et al., 2006; van Sluijs et al., 2021), and regular participation in PA is crucial for preventing various non-communicable diseases and improving and securing the sustainability of quality of life.

In alignment with the objectives outlined in Healthy China 2030, the national government implemented a comprehensive ecological strategy to improve PA and fitness levels among children and adolescents at the national level (Tan et al., 2017; Tan et al., 2019; Li, 2020). Understanding the correlates and potential mediating factors of health behaviors during childhood is crucial because this is a pivotal period for the establishment of lifelong healthy patterns. Thus, effective interventions should be designed for and implemented in this age group. During adolescence, obesity and related health issues can have long-term implications that extend to adulthood. This renders the establishment of PA patterns during adolescence a significant endeavor (Whitaker et al., 1997; Tremblay et al., 2012). Numerous studies have indicated that engaging children and adolescents in suitable physical activities can have positive effects on their physical movement functions (Malina, 2001; Comeras-Chueca et al., 2021). More specifically, prolonged involvement in PA can enhance physical coordination, flexibility, and balance in children and adolescents, consequently elevating their overall physical function (Battaglia et al., 2021). These findings underscore the significant advantages of active participation in physical activities for improving the physical movement functions of children and adolescents.

Within this context, in-depth examinations into the health behavior status of adolescents and the potential underlying causes may help us elucidate the factors impacting adolescent and children health. This could thereby broaden the avenues for non-pharmacological interventions aimed at enhancing health promotion.

Various physiological, psychosocial, familial, and environmental factors can influence PA behaviors in early life. As mentioned above, childhood is a crucial stage for establishing these behaviors, given the tendency of behaviors established during this period to persist into adulthood (Gustafson and Rhodes, 2006; Trost and Loprinzi, 2011), and family environment and caregiver influence are significant factors shaping children’s lifestyle habits (Yao and Rhodes, 2015; Khan et al., 2020). Social Learning theory suggested that children learn by observing and imitating their parents’ behaviors (Rumjaun and Narod, 2020). When parents actively participate in sports and show enthusiasm for exercise, children are more likely to mimic these behaviors (Sember et al., 2020). Furthermore, positive feedback and encouragement from parents can enhance children’s exercise behaviors, boost their self-confidence, and motivate them to engage in more physical activities (Hosokawa et al., 2023). In fact, there are studies showcasing the benefits of adequate and positive family functioning on PA levels and the mitigation of sedentary behavior in adolescents (Janssen et al., 2020).

A robust theoretical framework is provided by social cognitive theory, which suggests that cognitive, behavioral, and environmental factors collectively influence behavioral change. This framework has been widely recognized for its effectiveness in guiding the development of strategies aimed at enhancing physical activity levels, as evidenced by substantial supportive research (Young et al., 2014). There is also evidence demonstrating that parents exert a significant influence on their children’s PA levels, and frequently assume the principal responsibility for encouraging their children’s engagement in PA (Trost and Loprinzi, 2011; Su et al., 2022; Hosokawa et al., 2023). These studies collectively point to the cruciality of caregiver-related consciousness of healthy lifestyles in predicting the lifestyle habits of the next generation. In light of the potential influence of parents on their children’s PA, there is an urgent need to comprehensively understand this influence so that we can provide contextually-relevant reference data for guiding the development of effective strategies to enhance PA levels among children and adolescents. Related investigations also hold potential to provide insights to areas for future research.

Due to differences in physiological and psychological development, children of different ages and genders may respond differently to parental influence on physical activity. Parents’ attitudes, participation, and awareness regarding exercise have varying impacts on their children’s physical activity levels, influenced by age and gender. Research indicated that parents’ exercise consciousness exerted a more direct influence on girls, while for boys, this influence was often mediated through the role modeling of fathers (Ha et al., 2022). Age was recognized as an important factor in assessing the impact of parents on their children’s physical activity levels. Studies highlighted the influencing effects of age on the relationship between parental physical activity and the support and encouragement provided to children, which positively affected their engagement in sports and overall physical activity (Jago et al., 2014; Yao and Rhodes, 2015). Therefore, understanding these gender and age differences is essential for uncovering the mechanisms through which parents’ exercise consciousness affects children’s physical activity, providing a foundation for designing effective family and school-based physical activity interventions.

Enhancing parents’ comprehension of physical activity (PA) is pivotal for rectifying the common misperception of their children’s activity levels, often underestimating or overestimating their actual engagement in physical activities (Martínez-Andrés et al., 2020). By clarifying these perceptions, parents may be more inclined to motivate their children to increase PA, thereby improving overall health and fitness levels. It is well-established that there is a correlation between the PA levels of parents and their children, which interacts with factors such as age, gender, and intensity of activities (Petersen et al., 2020). Various parenting practices significantly influence children’s PA, as evidenced by research highlighting the impact of parental encouragement and support (Oliver et al., 2010; Zecevic et al., 2010), role modeling in PA (Smith et al., 2010; Hinkley et al., 2012; Rodrigues et al., 2018; Dgd et al., 2019; Hosokawa et al., 2023), and parents’ cognitions and identity related to their own and their children’s PA (Corder et al., 2012). Additionally, parental self-efficacy plays a crucial role in fostering a physically active lifestyle for children (The Lancet, 2019). General parenting styles that include verbal encouragement, logistical support, active engagement in PA with children, monitoring, and the use of reinforcement and rewards are effective strategies to promote PA (Lindsay et al., 2018). However, the cited studies have not individually examined the effects of each dimension on physical activity, instead focusing on overall outcomes. Understanding the psychological and behavioral characteristics of parents in supporting physical activity, and capturing the differences in cognitive, emotional, and behavioral domains (Timperio et al., 2013; Juriana et al., 2021), particularly regarding cognitive, attitudinal, and rights- and responsibility-related aspects, is crucial (Kovács et al., 2023). This emphasizes the need for further investigation into the predictive roles of different dimensions of parental exercise consciousness. By delving into these dimensions, we can better understand their potential impact on children’s participation in exercise.

Despite the wealth of research on individual aspects of parenting practices related to PA, there is a gap in understanding the combined and interactive effects of these variables. This gap underscores the necessity for further research to uncover how different dimensions of parental exercise consciousness—ranging from cognitive and attitudinal to rights- and responsibility-related aspects—can collectively influence and potentially enhance children’s PA levels. By exploring these dimensions, we can develop more targeted and effective interventions to boost physical activity among children, leveraging the significant role of parental influence.

Based on the above, and in an attempt to deliver supportive data for the development of effective strategies and potential approaches to address children’s PA insufficiency, such as by providing directions for enhancing parents’ understanding of the predictive associations of the various dimensions of parental exercise consciousness with their children’s PA status, we propose the following hypotheses.

Hypothesis 1: Children’s PA levels, intensity, and frequency significantly change with age, showing gender differences.

Hypothesis 2: Parental exercise consciousness positively associates with children’s PA overall, intensity, time and frequency.

Hypothesis 3: Different dimensions of parental exercise consciousness have varying associations with children’s PA.

## 2 Materials and methods

### 2.1 Participation

To ensure the representativeness and diversity of the sample, relevant statistical yearbooks were referenced in this study. Factors such as economy and geographical location were considered, leading to the selection of multiple schools, including those in urban centers, suburbs, and rural areas, as well as both key municipal schools and regular schools. Additionally, the development status of school infrastructure was included in the considerations to observe the impact of the school’s developmental background on children’s physical activity, thereby ensuring the broad applicability and in-depth understanding of the research results. To effectively elucidate the predictive association of parental exercise consciousness with children PA, 450 family units were from nine schools stratified by child grade and gender, and we recruited families of elementary school students from multiple schools of varying academic rankings from Tianjin, a city in the northern part of China. The study participants were recruited through collaboration with education authorities that have jurisdiction over affiliated schools, ensuring the distribution and collection of questionnaires. The questionnaire distribution and collection spanned from mid-March to early June in 2023. Participation in the survey was contingent on participants cohabiting with their parents, and the latter being their primary caregivers. Questionnaires were distributed to these families as part of the children’s homework assignments, and were collected as a part of these assignments; all questionnaires that were returned were thoroughly reviewed. We’ve crafted stringent inclusion and exclusion criteria, informed by past physical activity questionnaire studies and research related conditions, to ensure the reliability of our survey data (Hidding et al., 2018). Participants were required to be enrolled elementary students within a specific age range, free from significant health issues, able to complete the questionnaire, and voluntarily provide truthful responses with informed consent. Exclusions applied to those who had dropped out, were underage, lacked parental consent, had comprehension issues, special educational needs, severe mental health concerns, were in other studies, showed attention deficits, made logical errors, or incomplete responses (Guo et al., 2021). Questionnaires were completed anonymously with strict distribution and collection protocols, including a 5% random recheck and quality control measures to verify data authenticity. Extensive quality controls were in place, involving staff training, informed consent processes, assistance for younger respondents, designated collection and screening personnel, and data anonymization to safeguard privacy. All participants and their guardians provided informed consent.

From the initial 450 family units, only 401 completed the questionnaire, 40 questionnaires didn’t not meet the admission criteria and 361 family groups were included in the analyses.

### 2.2 Measures

#### 2.2.1 PA

The PA Rating Scale-3 (PARS-3) (Liang, 1994; Duan et al., 2022), was used to measure the PA of children in the past month. The scale’s internal consistency and reliability were 0.86 and 0.82, respectively, and it has three dimensions: duration, intensity, and frequency. Responses were assessed using a five-point Likert scale, and while the scale for exercise intensity and frequency ranged from 1 (light exercise) to 5 (vigorous exercise), that for duration ranged from 0 to 4. The total score range was 0–100 points. Regarding the assessment criteria for exercise volume, values less than 19 were classified as low, between 20 and 42 as moderate, and >43 as high volume.

#### 2.2.2 Parental exercise consciousness

The Chinese Citizens Exercise Consciousness Questionnaire developed in a prior study (Qiao, 2019) was used to investigate parental exercise consciousness (PEC) in the parents of primary school students.

The PEC scale comprises four dimensions. Cognition and Identity evaluates parents’ fundamental comprehension and identification with exercise, essential for the construction of exercise consciousness foundation (Juriana et al., 2021; Kovács et al., 2023). Sentiment and intention exposes their emotional engagement and intentions toward exercise, vital for participation (Furusa et al., 2021). Attitude and Willingness indicates parents’ inclination to motivate their children’s exercise involvement, a pivotal driver (Bonavolontà et al., 2021; Kovács et al., 2024). Rights and Responsibilities gauges the practical support parents offer, highlighting their significant role and responsibility in children’s exercise development (Rodrigues et al., 2018; Qunito Romani, 2020).

Exploratory factor analysis was used to identify the scale’s structure. We initially constructed a theoretical model for the scale and 32 items, which showed Cronbach’s alpha coefficients of 0.929 for the total scale and 0.883, 0.939, 0.762, and 0.757 for each dimension, respectively. Since all coefficients were above 0.7, the exploratory factor analysis results were considered consistent with the theoretical conceptualization, and the dimensions were deemed to have good reliability, homogeneity, and stability.

LISREL version 8.7 was used to perform a confirmatory factor analysis using maximum likelihood estimation for this scale. After removing eight items with factor loadings <0.5, 24 items remained, for which all factor loadings exceeded 0.5, and that contributed to a cumulative variance of 54.767%. The t-values of the estimated coefficients for the relationships between the latent and observed variables were significant at the 1% level. The main fit indices for the validated factor analysis between the latent and observed variables were generally within the desired range, indicating high reliability and validity for the modified scale, its consistency with theoretical expectations, and good convergent validity.

In this study, a five-point Likert scale ranging from 1 to 5 (fully consistent, mostly consistent, not satisfied, mostly inconsistent, fully inconsistent) was used to assess the degree of alignment between statement items and real-world experiences from respondents (Supplementary material). Higher composite scores indicated a higher parental exercise consciousness level.

#### 2.2.3 Statistical analysis

In this study, the formula used for calculating exercise volume was as follows:


ExerciseVolume=Intensity∗Time∗Frequency

The Chinese Citizens Exercise Consciousness Questionnaire score D was calculated using the following formula:


$$D=\left(y_{1}\omega_{1}/x_{1}+y_{2}\omega_{2}/x_{2}\ldots .+y_{n}\omega_{n}/x_{n}\right)/\sum \omega_{i}$$

where *D* is the parental individual standardized score for exercise consciousness; *y* is the score of a specific question item; *ω* is the comprehensive weight of a specific question item; *x* is the highest score of a specific question item; n is the sample size.

All data were calculated using SPSS version 19.0. Continuous variables with normal distributions were reported using means and standard deviations (SD), while participant characteristics (i.e., age, child gender, parent occupation and education level, and family income) were displayed by numbers and percentages. Descriptive statistics and analysis of variance (ANOVA) with post-hoc test analysis, were used to present the results for children’s PA based on age and gender. Correlation analysis was performed to investigate the association between parental exercise consciousness and children’s PA. Subsequently, correlation and hierarchical regression analyses were conducted to explore the roles played by gender and age (as covariates) in the relationship between these variables. The significance level was set at a *p* of 0.05.

## 3 Results

### 3.1 Basic information about the respondents

In total, 361 family groups were included, encompassing children aged 9–11 years. The children’s gender distribution was 55.68% for boys and 44.32% for girls. Additionally, family type (urban or rural), monthly income, and parental occupation and educational level were considered in the analysis (Table 1). The surveyed children were evenly distributed across three age groups, with a gender ratio pending slightly toward higher girl numbers. The survey indicated that 59.54% of the parental participants had higher education, predominantly held bachelor’s degrees, and that family income was primarily concentrated between 6,000 and 12,000 RMB per person in the family.

**Table 1:** Characteristics of participants.

Characteristic	*n*	%
Children (*n* = 361)		
Age		
10	102	28.25
11	118	32.69
12	141	39.06
Gender		
Female	201	55.68
Male	160	44.32
Parent		
Profession		
Laborer	60	16.60
Service Staff	39	10.80
Science, Education, Culture, Health and Professional Technicians	56	15.50
Enterprises, Institutions and Agencies Staff	56	15.50
Government Official	30	8.30
Self-employed	89	24.7
Others	31	8.60
Educational level		
Primary Education	59	16.34
Secondary Education	87	24.1
Associate Degree	91	25.21
Bachelor’s Degree	89	24.64
Master’s Degree	29	8.03
Doctoral Degree	6	1.66
Family income (RMB/Mouth)		
3,000 below	11	3.10
3,000–6,000	72	19.90
6,000–12,000	126	34.90
12,000–18,000	116	32.10
18,000 above	36	10.00

### 3.2 PA status of the child via age and gender

Table 2 shows the children’s PA characteristics. The prevalence of light PA (184 individuals, 51%) and moderate PA (169 individuals, 46.8%) was substantial, while vigorous PA was markedly less common (eight individuals, 8%).

**Table 2:** Students’ PA of various score distribution on age and gender.

Variant	Score	Age			Gender		Total
		10	11	12	Male	Female	
Children’s PA							
Intensity	1	6 (1.7%)	2 (0.6%)	5 (1.4%)	3 (0.8%)	10 (2.8%)	13 (3.6%)
	2	71 (19.7%)	77 (21.3%)	65 (18%)	83 (23%)	130 (36%)	213 (59%)
	3	25 (6.9%)	39 (10.8%)	70 (19.4%)	74 (20.5%)	60 (16.6%)	134 (37.1%)
	4	0 (0%)	0 (0%)	1 (0.3%)	0 (0%)	1 (0.3%)	1 (0.3%)
	5	–	–	–	–	–	–
	Total	102 (28.3%)	118 (32.7%)	141 (39.1%)	160 (44.3%)	201 (55.7%)	361 (100%)
	1	–	–	–	–	–	–
Time	2	52 (14.4%)	33 (9.1%)	28 (7.8%)	44 (12.2%)	69 (19.1%)	113 (31.3%)
	3	48 (13.3%)	80 (22.2%)	103 (28.5%)	108 (29.9%)	123 (34.1%)	231 (64%)
	4	2 (0.6%)	5 (1.4%)	10 (2.8%)	8 (2.2%)	9 (2.5%)	17 (4.7%)
	5	–	–	–	–	–	–
	Total	102 (28.3%)	118 (32.7%)	141 (39.1%)	160 (44.3%)	201 (55.7%)	361 (100%)
Frequency	1	–	–	–	–	–	–
	2	29 (8%)	14 (3.9%)	10 (2.8%)	18 (5%)	35 (9.7%)	53 (14.7%)
	3	56 (15.5%)	54 (15%)	72 (19.9%)	75 (20.8%)	107 (29.6%)	182 (50.4%)
	4	17 (4.7%)	41 (11.4%)	52 (14.4%)	58 (16.1%)	52 (14.4%)	110 (30.5%)
	5	0 (0%)	9 (2.5%)	7 (1.9%)	9 (2.5%)	7 (1.9%)	16 (4.4%)
	Total	102 (28.3%)	118 (32.7%)	141 (39.1%)	160 (44.3%)	201 (55.7%)	361 (100%)
PA level	LPA	78 (21.6%)	52 (14.4%)	54 (15%)	65 (18%)	119 (33%)	184 (51%)
	MPA	24 (6.6%)	66 (18.3%)	79 (21.9%)	91 (25.2%)	78 (21.6%)	169 (46.8%)
	VPA	0 (0%)	0 (0%)	8 (2.2%)	4 (1.1%)	4 (1.1%)	8 (2.2%)
	Total	102 (28.3%)	118 (32.7%)	141 (39.1%)	160 (44.3%)	201 (55.7%)	361 (100%)

Regarding PA intensity, the distribution was skewed toward the lower end, with scores ranging from a minimum of 1 to a maximum of 4, and a modal score of 2. The duration was similarly skewed, with a minimum score of 2, a maximum of 4, and a modal score of 3, indicating relatively brief engagement periods. Frequency exhibited a broad range, from a minimum score of 2 to a maximum of 5, and a modal score of 3, denoting a trend toward regular participation (Table 2).

To elucidate potential differences in PA levels across age and gender, a least significant difference post-hoc analysis was conducted subsequent to a preliminary ANOVA. As depicted in Table 3, age and gender appeared to significantly associate with children’s PA scores (*p* < 0.001). There were significant differences across age groups regarding PA intensity, duration, and frequency (*p* < 0.01), whereas significant differences in relation to gender were observed only for intensity (*p* < 0.001) and frequency (*p* = 0.009). The above findings support Hypothesis 1, suggesting that the PA levels of the 9–11 age group increase with age, and that there are differences in activity patterns by gender, reflecting varying elements of PA status.

**Table 3:** Descriptive analysis and AVONE tests analysis of children’s PA score.

			Descriptive analysis					AVONE and LSD tests analysis		
Variant			*N*	Mean ± SD	95%CI	Min	Max	*F*	Sig	LSD
PA level	Age	9	102	16.19 ± 7.11	14.79–17.58	4	36	30.52	<0.001***	11 > 10 > 9
		10	118	21.33 ± 6.84	20.08–22.58	8	36			
		11	141	24.77 ± 10.36	23.05–26.50	4	64			
	Gender	M	160	23.15 ± 9.06	21.74–24.56	4	48	13.27	<0.001***	–
		F	201	19.69 ± 8.90	18.45–20.92	4	64			
	Total		361	21.22 ± 9.13	20.28–22.17	4	64	–	–	–
	Age	9	102	2.19 ± 0.52	2.08–2.29	1	3	8.72	<0.001***	11 > 10,9
Intensity		10	118	2.31 ± 0.50	2.22–2.40	1	3			
		11	141	2.48 ± 0.58	2.38–2.57	1	4			
	Gender	M	160	2.44 ± 0.54	2.36–2.53	1	3	10.33	0.01**	–
		F	201	2.26 ± 0.55	2.18–2.34	1	4			
	Total			2.34 ± 0.55	2.28–2.40	1	4	–	–	–
Time	Age	9	102	2.51 ± 0.54	2.40–2.62	2	4	14.69	<0.001***	11,10 > 9
		10	118	2.76 ± 0.52	2.67–2.86	2	4			
		11	141	2.87 ± 0.51	2.79–2.96	2	4			
	Gender	M	160	2.78 ± 0.53	2.69–2.86	2	4	1.66	0.198	–
		F	201	2.70 ± 0.55	2.63–2.78	2	4			
	Total			2.73 ± 0.54	2.68–2.79	2	4	–	–	–
Frequency	Age	9	102	2.88 ± 0.66	2.75–3.01	2	4	18.14	<0.001***	11,10 > 9
		10	118	3.38 ± 0.80	3.24–3.53	2	5			
		11	141	3.40 ± 0.70	3.28–3.51	2	5			
	Gender	M	160	3.36 ± 0.76	3.24–3.48	2	5	6.90	0.009***	–
		F	201	3.15 ± 0.74	3.05–3.26	2	5			
	Total			3.25 ± 0.76	3.17–3.32	2	5	–	–	–

**p < 0.01, ***p < 0.001.

### 3.3 Parental exercise consciousness

Table 4 shows the results for parental exercise consciousness. The composite metric for parental exercise consciousness spanned from a minimum of 50.59 to a maximum of 94.32, and showed a modal value of 86.23, suggesting a concentration in the upper-mid echelon. The scores across the four dimensions were uniformly elevated, indicating that the parental exercise consciousness resides in the upper-middle tier, with a harmonious distribution across the dimensions (Table 4).

**Table 4:** Descriptive analysis of parental exercise consciousness among various dimensions.

	Mean	SD	Min	Max	Median	Mode
PEC	79.45	8.40	50.59	94.32	82.51	86.23
CI	4.16	0.48	3.00	4.88	4.38	4.50
SI	3.91	0.46	2.29	4.86	4.00	4.14
AW	3.81	0.51	2.00	5.00	4.00	4.00
RR	4.14	0.56	3.00	5.00	4.00	4.00

PEC, Parental Exercise Consciousness; CI, cognition and identity; SI, sentiment and intention; AW, attitude and willingness; RR, rights and responsibilities.

### 3.4 Hierarchical regression analysis of parental exercise consciousness and children’s PA

Table 5 shows the Pearson correlation analysis results for the association of parental exercise consciousness and children’s PA. The analysis revealed a correlation coefficient (*r*) of 0.601, denoting a significantly moderate and positive linkage between the constructs of interest (*p* < 0.05).

**Table 5:** Correlation between children’s PA level and PEC.

	1	2	3	4	5	6	7	8	9
1. Age	1								
2. Gender	−0.169**	1							
3. Intensity	0.215**	−0.167**	1						
4. Time	0.268**	−0.068	0.222**	1					
5. Frequency	0.264**	−0.137**	0.051	0.196**	1				
6. CI	0.295**	−0.155**	0.489**	0.481**	0.387**	1			
7. SI	0.329**	−0.178**	0.475**	0.547**	0.479**	0.784**	1		
8. AW	0.307**	−0.162**	0.554**	0.425**	0.358**	0.678**	0.743**	1	
9. RR	0.282**	−0.153**	0.436**	0.328**	0.348**	0.665**	0.568**	0.576**	1

**p < 0.01.

We also assessed the latent associations between the dimensions of parental exercise consciousness and children’s PA. There were moderately positive correlations between two dimensions of parental exercise consciousness (cognition and identity and sentiment and intention) with children’s PA intensity, duration, and frequency (cognition and identity simple correlation coefficients: 0.489, 0.481, and 0.387, respectively; sentiment and intention simple correlation coefficients: 0.475, 0.547, and 0.479, respectively). Meanwhile, the dimensions of attitude and willingness and rights and responsibilities exhibited moderately positive correlations with children’s PA intensity and duration (attitude and willingness simple correlation coefficients: 0.554 and 0.425, respectively; rights and responsibilities: 0.436 and 0.328, respectively), and weak positive correlations with frequency (attitude and willingness: 0.358; rights and responsibilities: 0.348). All of these correlations showed a *p* < 0.05, indicating some moderate and some weak correlations between the dimensions of parental exercise consciousness and children’s PA (Table 5).

All four models showed a *p* < 0.001, indicating that gender and age significantly associated with children’s PA and its components. Furthermore, the explanatory variables accounted for 37% of the variance in PA. The variance inflation factors were below five, suggesting minimal multicollinearity among the variables (Table 6).

**Table 6:** Hierarchical regression analysis of children’s PA.

		Mode1	Mode2	Mode3	Mode4
Intensity	CI	0.168	0.169	0.168	0.168
	SI	0.017	0.008	0.012	0.005
	AW	0.410***	0.407***	0.407***	0.405***
	RR	0.111	0.107	0.109	0.105
	Gender	/	−0.073	/	−0.071
	Age	/	/	0.015	0.010
	F	45.281***	36.801***	36.191***	30.606***
	R2	0.337	0.341	0.338	0.342
	Adj-R2	0.337	0.332	0.000	0.330
Time	CI	0.163	0.162	0.162	0.161
	SI	0.512***	0.517***	0.491***	0.496***
	AW	0.022	0.024	0.012	0.013
	RR	−0.025	−0.023	−0.036	−0.034
	Gender	/	0.035	/	0.046
	Age	/	/	0.064*	0.067*
	F	39.309***	31.504***	32.572***	27.285***
	R2	0.306	0.307	0.314	0.316
	Adj-R2	0.299	0.298	0.305	0.305
Frequency	CI	−0.061	−0.06	−0.062	−0.062
	SI	0.760***	0.751***	0.726***	0.720***
	AW	−0.048	−0.051	−0.064	−0.066
	RR	0.179*	0.174*	0.161	0.158
	Gender	/	−0.074	/	−0.057
	Age	/	/	0.103*	0.099*
	F	27.972***	22.596***	23.659***	19.801***
	R2	0.239	0.241	0.25	0.251
	Adj-R2	0.231	0.231	0.239	0.239

**p < 0.01, ***p < 0.001.

PEC, Parental Exercise Consciousness; CI, cognition and identity; SI, sentiment and intention; AW, attitude and willingness; RR, rights and responsibilities.

We further investigated the correlations of the four dimensions of parental exercise consciousness with children’s PA intensity. Results showed that without considering the covariates of gender and age, cognition and identity, sentiment and intention, and rights and responsibilities did not significantly associate with children’s PA intensity (*p* > 0.05). Meanwhile, considering the other variables constant, a one-point increase in attitude and willingness correlates with a 0.41-point increase in children’s PA intensity (*p* < 0.05); this significance remains unchanged even after introducing gender and age as control variables, albeit the increase in intensity goes from 0.410 to 0.405 (Table 6). These results validate Hypothesis 2.

Regarding the regressions for children’s PA duration, without considering the covariates (gender and age), cognition and identity, attitude and willingness, and rights and responsibilities showed non-significant correlations with PA duration (*p* > 0.05). Conversely, sentiment and intention correlated significantly with PA duration (*p* < 0.05); specifically, a one-point increase in sentiment and intention associates with a 0.512-point increase in PA duration. The inclusion of gender and age does not alter the significance of this association, but the increase goes from 0.512 to 0.496 (Table 6).

Regarding the regressions for children’s PA frequency, they were generally consistent with those for intensity. Maintaining consistency across all other variables, a one-unit increment in this aspect results in a 0.760-point elevation in the overall score for PA duration. With the progressive integration of gender and age as control factors, the relevance of this dimension remained unchanged; however, its effect diminished from 0.760 to 0.720 (Table 6). These findings validate Hypothesis 3.

## 4 Discussion

This study aimed to investigate the correlation between parental exercise consciousness and children’s PA characteristics (i.e., duration, frequency, and intensity) across different grades and genders within a family context and among children aged 9–11 years. In so doing, it addresses questions regarding the potential underlying mechanisms, associations, and pathways of the relation, and hence contributes to sustainable social development. This study found notable differences in children’s PA based on age and gender, and there was a moderate correlation between parental exercise consciousness and various aspects of children’s PA, supporting Hypothesis 1.

The findings also underscore the asymmetrical association between parental exercise consciousness and children’s PA. It seems that enhancements in parental consciousness of exercise-related emotions, intentions, attitudes, and willingness are effectively and predictively associated with the intensity, duration, and frequency of children’s PA. These findings align with hypotheses 2 and 3.

### 4.1 PA and children’s demographic characteristics

We observed a predominance of light to moderate PA within the sample, and the children’s PA was characterized by a tendency toward lower intensity, duration, and a moderately high frequency of engagement. Age and gender had differential associations with children’s PA total score, duration, intensity, and frequency. These pieces of evidence correspond with those reported in related research in China (Chen et al., 2021; Yang et al., 2022). Furthermore, various studies have revealed that girls exhibit lower perceived competence in physical education and are less active and physically fit than boys (Ishii et al., 2015; Telford et al., 2016). A cross-sectional observational study on the PA of 248 children aged 7.9–11.1 years demonstrated that older and male participants engaged in higher amounts of daily PA (Dencker et al., 2006). There were also gender differences in PA with increasing age among 632 parent dyads of children and adolescents from Ethiopia (Biadgilign et al., 2022). A Canadian study involving 1,057 children of normal weight reaffirmed this trend, with boys in grades 3, 7, and 11 dedicating 9, 22, and 27%, respectively, more time to moderate-to-vigorous PA than their female counterparts (Thompson et al., 2005). A study on youth PA also supports our findings, showing that male individuals exhibit higher levels of moderate PA and vigorous PA across most age groups ranging from 8 to 13 years (Sherar et al., 2007). These previous studies lend empirical support to the gender and developmental trends in PA captured by the findings of this investigation.

Notably, in this study, children’s PA intensity, duration, frequency, and parental exercise consciousness did not decline with age, which is inconsistent with previous findings. With the exception of the children’s PA duration and intensity, the 11-year-old cohort exhibited significantly higher levels than the 9-year-old cohort across all PA parameters. Among the three intergroup variances, only the intensity and duration exhibited notable changes. These findings suggest disparities in PA with the primary results of similar past studies, which have shown that the decline in PA is most pronounced at puberty—which generally begins at age 13 years (Mitchell et al., 2012). It is important to note that while our survey was administered during the academic term, the survey questionnaire did not distinguish between extracurricular PA and school physical education. Consequently, it was not feasible to exclude the potential association of curriculum complexity—and its variations by grade—on the observed PA patterns. Still, future studies could further probe into whether the evolving physical education syllabus across grades accounts for the lack of a discernible decline in PA with increasing age among students.

Regarding gender disparities, they predominantly lied in children’s PA intensity and frequency. In particular, boys exhibited higher levels of moderate-to-vigorous PA intensity and frequency than girls (Table 3). The majority of the existing literature corroborates our findings on gender disparities for PA intensity, but way fewer corroborate the results for frequency. For example, the degree of gender-related variation depends on the character and rigor of the activity—which is consistent with our results—suggesting that the dissimilarity in PA levels between boys and girls arises predominantly from divergences in intensity rather than PA duration or frequency (Telama and Yang, 2000; Klinker et al., 2014). Research on domain-specific PA in children has identified substantial gender disparities, with girls displaying lower levels of engagement across nearly all domains and subdomains (Sun et al., 2023). In summary, PA levels were notably higher in boys than in girls, boys showed predominantly higher PA intensity and frequency, there were non-significant variances observed for duration, and these results partly corroborate the available evidence in the literature.

### 4.2 The predictive role of parental exercise consciousness on children’s PA

We observed a correlation of parental exercise consciousness with PA intensity, frequency, and duration among 9–11-year-old children of both genders. Distinct components of parental exercise consciousness differentially associated with the constituents of PA, with attitude and willingness significantly correlating with intensity, while sentiment and intention had a more pronounced correlation with duration and frequency. Specifically, the enhancement of attitude and willingness seems to associate with the promotion of a more intrinsic motivation to engage in physically-intense activities (Bonavolontà et al., 2021; Kovács et al., 2024), whereas the cultivation of sentiment and intention may associate with encouragement for longer and more frequent bouts of PA through social support and modeling behaviors (Furusa et al., 2021). These findings suggest that parental exercise consciousness may possess a predictive association with the PA of children within the age groups under scrutiny; we can thus infer that enhancing specific content areas such as parental consciousness toward exercise-related attitudes, willingness, sentiments, and intentions could support improvements in children’s PA. In showcasing these findings, this research contributes to the burgeoning field of pediatric exercise promotion by elucidating the associative role of parental exercise consciousness with the PA profiles of children and preadolescents. This study offers empirical evidence to support the development of family-based interventions focusing on enhancing parental consciousness of and involvement in their children’s PA. Such interventions could leverage the predictive power of parental exercise consciousness to create an environment conducive to the adoption and maintenance of active lifestyles during childhood and adolescence.

From a socio-ecological perspective, individual behavior is shaped by numerous factors and their interplay, such as organizational structure, surrounding environment, interpersonal social connections, and the individual (Young et al., 2014; Hu et al., 2021; Cutrín et al., 2022). The roles that caregivers play on individual behavior also cannot be overlooked, as they are fundamental and significant in shaping a child’s development. Still, knowledge continues lacking about the impact of parents on children’s PA, as previous studies have primarily concentrated on parental attitudes and behaviors and overlooked the underlying determinants of parental actions (Mitchell et al., 2012). Therefore, the focus of the current study was to understand the predictive pathways of the association between parental exercise consciousness and children’s PA. We observed an overall level of parental exercise consciousness in the upper middle class, with its dimensions (i.e., cognition and identity, rights and responsibilities, emotions and intentions, and attitudes and willingness) showing relatively balanced levels in this class.

In a past systematic review on the correlation between parents and the PA levels of children aged 6–12 years, parents significantly influenced children’s PA, and the significance of promoting children’s PA within the home setting was underpinned (Zecevic et al., 2010). Parental support has also been shown to exert a positive impact on children’s PA level by inducing intrinsic motivational changes when significant alterations in PA level take place (Petersen et al., 2020; Liu et al., 2023). A study assessing PA interventions aimed at young children determined that successful obesity prevention programs targeting early childhood should be grounded in a comprehensive understanding of how specific parental beliefs and behaviors impact various aspects of the obesogenic system (Mado et al., 2021). It is thus imperative to implement effective interventions that encompass various parental factors at multiple levels to sustain or enhance PA in children and adolescents.

A study was conducted with 61,429 children between ages 6–18 years along with their parents in Shanghai, China, and indicated that diverse forms of parental assistance exert a noteworthy influence on moderate-to-vigorous PA in children and adolescents (Hong et al., 2020). Furthermore, these effects appeared to differ according to gender and grade. In our study, sentiment and intention, attitude and determination, and a positive perspective toward children’s PA, exhibit significant associations with gender and age. These results are partly consistent with those in previous studies focused on parental attitudes toward and involvement in children’s PA (Bentley et al., 2012; Kesten et al., 2015). There are also studies showing that parents’ perceptions of their children’s health behaviors (e.g., underestimation of PA) impact their children’s behaviors. For example, research on mothers’ perceptions of their children’s weight development revealed a connection between mothers underestimating their children’s weight and an increase in weight development, highlighting the importance of supporting parents in developing more realistic perceptions of health consciousness and behaviors (Vrijkotte et al., 2020). This conclusion partially aligns with the findings of the current study, highlighting the enduring predictive association between parental exercise consciousness and the sustained PA levels of children. Nonetheless, there is a lack of pertinent research that can substantiate the impact of parents’ rights and responsibilities regarding exercise on children’s PA, possibly due to cognitive variances in this subject across different countries.

We also observed the positive association of the dimensions of parental exercise consciousness of cognition and identity, and, sentiment and intention with the three dimensions of children’s PA (i.e., intensity, duration, and frequency; Tables 5, 6). Meanwhile, the dimensions of attitude and willingness, and, rights and responsibilities exhibited a stronger positive association with PA intensity and duration than with frequency. That is, there were differential findings regarding the relation different dimensions of parental exercise consciousness with children’s PA. To comprehend the mechanisms through which age and gender contribute to the positive predictive association between parental exercise consciousness on children’s PA, we conducted stratified linear regression analyses. It indicated that at least one factor, either gender or age, contributes to this association.

The findings of correlation tests and regression analyses revealed a positive correlation between multiple dimensions of parental exercise consciousness and children’s PA intensity, and that parental exercise consciousness holds a significant predictive association with children’s PA intensity. When researchers scientifically assess PA for its effectiveness and impact on health, exercise intensity is often considered a core element of the measurement process. Indeed, appropriate exercise intensity not only enhances the effectiveness of exercise but also serves as a direct means of achieving significant health benefits (Saunders et al., 2016). Therefore, parents’ profound understanding and positive attitudes toward PA may positively associate with their children’s exercise intensity, thereby exerting a positive influence on their children’s health and physical fitness. This study emphasizes the importance of parental exercise consciousness in encouraging children to actively engage in PA, delivering a theoretical basis for the future design and implementation of effective PA interventions in home environments.

However, PA intensity is often coupled with the inherent risk of injury and the reality thereof (Kellmann, 2010). During high-intensity workouts, the increased physiological demands on the body can increase the likelihood of injury, emphasizing the need to balance the pursuit of health benefits with the inherent risks associated with strenuous physical activities. Consequently, a nuanced understanding of exercise intensity is essential for developing safe and effective PA programs that minimize injury incidence while maximizing health outcomes. In pediatric PA, the risk of injury is a primary concern for parents; accordingly, as their children engage in sports, parents frequently admonish them to exercise caution and slow down to mitigate potential injury risks. However, trepidation toward injury and an overly cautious stance may inadvertently influence parental control over the intensity of their children’s physical activities (Hamstra et al., 2002). These descriptions showcase how parental understanding of appropriate exercise intensity directly shapes the patterns of their children’s physical behavior, thereby affecting the development of PA habits and their quality. Therefore, the depth of parents’ scientific understanding of exercise intensity is crucial for devising sensible exercise plans for children, ensuring safety in physical activities, and fostering healthy growth. The takeaway here is that equipping parents with accurate scientific knowledge regarding exercise prescription is key to optimize the benefits of PA for their children while minimizing the risks of injury.

In a survey on parental perspectives about sports-related injuries in their children, over 80% of the 1,000 parents of adolescent athletes were not acquainted with guidelines pertinent to adolescent engagement in sports (Bell et al., 2020). This prevalent lack of awareness may engender a psychological predisposition rooted in concerns about their children’s safety, potentially skewing their approach to professionalizing their children’s training regimens. Based on the findings of this cited study, enhancing parents’ understanding of exercise science positively influences the intensity of their children’s PA levels, thereby improving the health benefits of PA for children. Specifically, parents well informed about the science of exercise are more likely to guide and encourage their children to engage in physical activities of moderate intensity. As aforementioned, this approach may ensure safety while maximizing the health benefits of exercise. This shows the importance of educational and intervention initiatives aimed at increasing parental knowledge in this area for fostering positive PA habits and enhancing children’s overall health status. Furthermore, within the domain of familial health enhancement, PA augmentation extends its relevance beyond the general population, offering significant benefits for motor capability and social functionality advancement in individuals with neurodevelopmental disabilities, including those with autism spectrum disorder. For instance, empirical case reports have indicated that tailored exercise modalities can markedly influence gross motor skill development and social conduct in children with autism (Battaglia et al., 2019). Consequently, the informed engagement of caregivers in such practices may constitute a viable and effective strategy for ameliorating the physical and social dynamics of children.

Our correlational findings highlighted those parental emotions, intentions, attitudes, and willingness—within the context of parental exercise consciousness—may be pivotal predictive factors associating with children’s PA. Consequently, targeted educational interventions aimed at parents can elevate their comprehension of the significance of sports activities for their children, incite affirmative emotional responses and a sense of voluntariness, and cultivate an optimistic and initiative-oriented stance toward physical engagement. In summary, encouraging shared sports activities between parents and children may engender affirmative parent–child sports-related interactions, augmenting familial awareness and involvement in such activities, which could yield beneficial outcomes for children’s PA. Enriched by Social Learning Theory, the investigation delved into the influence of parental exercise consciousness on children’s physical activity behavior, offering fresh insights into health promotion. The study unveiled the mechanisms that enable parents’ exercise consciousness to enhance adolescents’ engagement in physical activity. Factors such as gender and age were also examined to deepen the understanding of how parental exercise consciousness shapes children’s physical activity in everyday life.

## 5 Limitations and strengths

First, the study’s limited and demographically specific sample size, coupled with the exclusive focus on Tianjin, may impede the generalizability of the findings to other regions, given the diverse economic, cultural, and environmental factors that can influence children’s physical activity levels nationwide. Second, while the survey methodology used was adaptable, the study could be strengthened in future research by incorporating accelerometers to collect more robust empirical data. Third, the study did not adequately consider the effects of temperature variations across different seasons on physical activity, which could limit the interpretive power of the results. Future studies could address this limitation by incorporating seasonal data analysis, which would allow for a more nuanced understanding of how weather impacts physical activity levels throughout the year. Despite these limitations, the research nonetheless provides a foundation for subsequent inquiries into the interplay between familial determinants and children’s physical activity, enhancing our understanding of the factors that shape children’s health behaviors.

## 6 Conclusion

This study explored the predictive association of parental exercise consciousness with the PA levels of 9–11-year-old children, highlighting age and gender differences. It revealed an increasing trend in children’s PA levels in terms of time, intensity, and frequency as age advanced, with boys exhibiting higher levels of PA intensity than girls in the age group of 9–11 years. The results showed that parental exercise consciousness positively correlated with children’s PA behaviors, with sentiments, intentions, attitudes, and willingness playing significant roles. Enhancing parental exercise consciousness can boost children’s PA engagement, and understanding the parental influence on children’s PA is crucial for developing effective strategies. Family environment significantly shapes children’s lifestyle habits, emphasizing the importance of parental involvement in promoting PA. Overall, this study provides insights into how parental consciousness and involvement can influence children’s physical behaviors over time.

## Data Availability

The original contributions presented in the study are included in the article/Supplementary material, further inquiries can be directed to the corresponding author.
