# Supplementary material for: Enhancing physical activity levels in 9–11-year-old children of varied genders: strategies leveraging parental exercise consciousness
Source: Front Psychol. 2025 Feb 6;16:1407090. doi: 10.3389/fpsyg.2025.1407090 (PMC11839719; doi:10.3389/fpsyg.2025.1407090)
Supplement: Supplementary file 1 [file Table_1.DOCX]

Supplementary Material

Table 1. Contents and weight of Chinese Citizens Exercise Consciousness Questionnaire

| **Dimension** | **Weight** | | | **Items** |
| --- | --- | --- | --- | --- |
| Cognition  and  Identity | 0.28 | 0.45 | 0.30 | 1 I strongly agree with the value and function of physical activity for fitness, mindfulness and beauty. |
|  |  |  | 0.26 | 2. I strongly recognize the role of physical activity in promoting the all-round development of human beings and enhancing the sense of well-being. |
|  |  |  | 0.24 | 3. I believe that physical activity is a good medicine that is effective in preventing and treating diseases. |
|  |  |  | 0.20 | 4. I am fully aware of the dangers of physical inactivity and its relationship to chronic diseases. |
|  |  | 0.55 | 0.22 | 5. I have a basic knowledge of physical health care |
|  |  |  | 0.28 | 6. I have a very good understanding of the principles, methods and prescription of PA. |
|  |  |  | 0.24 | 7. I have a good understanding of the principles of PA. |
|  |  |  | 0.26 | 8. I have a good understanding of the safest and most effective types and methods of PA. |
| Sentiment  and  Intention | 0.34 | 0.55 | 0.20 | 9. I've already experienced the benefits of PA. |
|  |  |  | 0.30 | 10.I get excited when I think about doing exercise |
|  |  |  | 0.24 | 11.I'm eager to do exercise, it's a pleasure for me Every time I do exercise, I feel very happy |
|  |  |  | 0.26 | 12. I want to make PA a priority in my daily life |
|  |  | 0.45 | 0.30 | 13. I intend to be physically active at least 3 times a week for at least 30 minutes in the next 6 months. |
|  |  |  | 0.35 | 14. I am willing to spend time, energy and money on PA. |
|  |  |  | 0.35 | 15. I am very willing to join exercise associations, clubs and other public exercise organizations. |
| Attitude  and  Willingness | 0.26 | 0.50 | 0.30 | 16. Even if I am busy at work, I willing still find some time to participate in PA. |
|  |  |  | 0.35 | 17. I always try to maintain or improve my health through various forms of exercise. |
|  |  |  | 0.35 | 18. No matter how difficult it is, I've been doing it for at least half a year. |
|  |  | 0.50 | 0.55 | 19. Despite the fact that it is very hard and tiring to exercise, I always stick to my exercise program. |
|  |  |  | 0.45 | 20. I believe that I have the right to share the fruits of exercise development. |
| Rights  and  Responsibilities | 0.12 | 0.50 | 0.50 | 21. I believe that I have the right to enjoy public exercise services. |
|  |  |  | 0.50 | 22. I believe that I have the responsibility to fulfill the obligations of the National Fitness Program. |
|  |  | 0.50 | 0.45 | 23. I believe that when I improve my physical fitness level through physical activity, I also contribute to building a "Health for All" society. |
|  |  |  | 0.55 | 24.I believe that when we improve our physical fitness level through physical activity, we also take responsibility for building a healthy society for all. |
